# A decade of 10-valent pneumococcal conjugate vaccine use in Lithuania: trends in invasive pneumococcal serotype dynamics

**DOI:** 10.3389/fpubh.2025.1633396

**Published:** 2025-08-01

**Authors:** Aurelija Petrutienė, Jekaterina Sinotova, Nijolė Pupienienė, Raminta Marcinonytė, Indrė Padvilikytė, Jelena Razmuk, Svajūnė Muralytė, Aistė Bulavaitė, Milda Plečkaitytė

**Affiliations:** ^1^Department of Bacteriology, National Public Health Surveillance Laboratory, Vilnius, Lithuania; ^2^Department of Molecular Biology, National Public Health Surveillance Laboratory, Vilnius, Lithuania; ^3^Institute of Biotechnology, Life Sciences Center, Vilnius University, Vilnius, Lithuania

**Keywords:** *Streptococcus pneumoniae*, invasive pneumococcal disease, serotype, pneumococcal conjugate vaccine, serotype replacement, surveillance

## Abstract

**Background:**

*Streptococcus pneumoniae* causes invasive pneumococcal disease (IPD), a serious condition characterized by the spread of pneumococci to normally sterile human body sites. Pneumococcal conjugate vaccines (PCVs) have reduced IPD incidence caused by vaccine serotypes, though non-vaccine serotypes remain a risk. Lithuania introduced the 10-valent PCV (PCV10) into the National Immunization Program in 2014, with a subsequent switch to PCV15 in 2024. This study aimed to assess the impact of PCV10 on the distribution of invasive serotypes across the population and age groups over a decade of vaccine implementation.

**Materials and methods:**

A total of 1,190 invasive pneumococcal isolates collected during the pre-PCV10 (2010–2014) and post-PCV10 (2015–2024) periods were stored at the National Public Health Surveillance Laboratory and routinely serotyped using the Quellung reaction and multiplex PCR. We analyzed serotype distribution in the overall population and separately in children and adults, with the adult group further stratified into 18–64 and ≥65 years.

**Results:**

The number of invasive pneumococcal isolates significantly exceeded the annually reported IPD cases, indicating substantial underreporting in Lithuania. The proportion of PCV10 serotypes declined significantly in the overall population, decreasing from 50% in 2010–2014 to 20% in 2024 (*p* = 0.00002) and within age-specific groups. Non-PCV10 serotypes, primarily 19A (*p* = 0.0015), 3 (*p* = 0.004), and 6C (*p* = 0.0061), and serotypes 8 and 22F, showed increasing trends. Serotype 3 has remained the most prevalent IPD serotype since 2015. From 2018 onward, serotype 19A became the second most common serotype among adults aged 18–64 years, while its increase among children was less apparent, likely due to the low number of pediatric isolates.

**Conclusion:**

This is the first study in Lithuania to demonstrate that the childhood vaccination program reduced IPD caused by vaccine serotypes in children and unvaccinated adults through indirect protection. However, serotype replacement following PCV10 introduction likely contributed to the observed increase in non-vaccine serotype IPD cases among adults. Limitations in current IPD surveillance hinder the ability of Lithuanian health authorities to make timely, evidence-based decisions regarding the impact of PCVs. Strengthening surveillance systems is essential to inform and guide effective pneumococcal vaccination strategies.

## 1 Introduction

A gram-positive bacterium, *Streptococcus pneumoniae* (pneumococcus), is a common cause of bacteremic pneumonia, meningitis, and sepsis, collectively termed invasive pneumococcal disease (IPD) (1). Pneumococci are a significant cause of morbidity and mortality worldwide, particularly in children and older adults (2, 3). The primary virulence factor of pneumococci is the polysaccharide capsule (1). More than 100 capsular serotypes have been described, although a subset is involved in the development of IPD. Disease prevention is realized using pneumococcal conjugate vaccines (PCVs), which are based on capsular polysaccharides as the antigen (4). PCVs cover an array of serotypes selected according to their contribution to pneumococcal infections. The introduction of PCVs into routine childhood immunization programs has strongly reduced overall and vaccine-type pneumococcal disease incidence in many countries (4). However, the reduction in certain vaccine-covered serotypes is often counteracted by increases in non-vaccine serotypes, a phenomenon known as serotype replacement (5, 6). As a result, non-vaccine serotypes occupy the ecological niche of human nasopharyngeal microbiota, becoming more prevalent in the population. Therefore, surveillance of serotype distribution before vaccine implementation and ongoing serotype replacement monitoring in the post-vaccine period is highly recommended, considering the specific characteristics of each country or region.

In Lithuania, PCV targeting 10 pneumococcal serotypes (PCV10) was introduced into the Lithuanian National Immunization Program (NIP) on October 1, 2014, using a 2+1 infant schedule with doses administered at 2, 4, and at 12–15 months of age. Within the NIP, PCV targeting 13 serotypes (PCV13) has been administered to at-risk children over the age of 2 who have not previously received other PCVs, as well as to at-risk adults. Outside the NIP, adults aged over 65 years had the option to be vaccinated with pneumococcal polysaccharide vaccine PPV23. In 2024, the NIP was updated, replacing the PCV10 vaccine with PCV15 for childhood vaccination. At-risk children and at-risk adults (including individuals aged over 75 years) are now vaccinated with PCV20 instead of PCV13 within NIP.

Currently, there are no data on the impact of vaccination on pneumococcal disease in children and adults, including serotype distribution in Lithuania, information that is crucial for shaping national vaccine strategies. Lithuania does not participate in any international programs and initiatives for IPD, including those focused on genetic characterization and surveillance (7, 8). A single laboratory-based surveillance study performed in 2012–2013 in Lithuania, before the implementation of PCV10 into the NIP, found pneumococcal carriage prevalence of 40% in children under 6 years of age with acute upper respiratory tract infection, and serotypes 6B, 19F, and 23F were most frequently isolated (9).

To address this gap and provide evidence-based guidance for future vaccination strategies, we aimed to assess the impact of PCV10 on the distribution of invasive pneumococcal disease serotypes in Lithuania across the overall population and specific age groups, covering the pre-vaccination period (2010–2014) and the post-vaccination period (2015–2024).

## 2 Materials and methods

### 2.1 Study design, sources of isolates and data

The retrospective study on invasive *S. pneumoniae* isolates covered a 5-year pre-PCV10 period (2010–2014) and a 10-year post-PCV10 period (2015–2024). These periods are also referred to in the text as the pre-vaccine and post-vaccine periods, respectively. Although the NIP was updated in July 2024 to replace PCV10, the transition in Lithuania has occurred gradually. According to the Ministry of Health directive, the switch to PCV15 will be implemented once all existing PCV10 doses have been administered. Some healthcare providers, particularly those in smaller towns, maintained reserves of PCV10, thereby prolonging the transition period. To account for this variability, we defined the post-PCV10 vaccine period as ending December 31, 2024.

In Lithuania, all cases of IPD are subject to mandatory reporting. Since 2009, invasive *S. pneumoniae* isolates have been required to be submitted to the National Public Health Surveillance Laboratory (NPHSL) (10). During the study period, NPHSL received invasive pneumococcal isolates from laboratories of secondary and tertiary hospitals and independently operating certified laboratories authorized to identify *S. pneumoniae* in human samples. These hospitals and laboratories serve Lithuania's 2.8 million population. The laboratories initially identified pneumococcal isolates using conventional microbiological methods and/or Matrix-assisted laser desorption/ionization time-of-flight (MALDI-TOF) mass spectrometry.

At the NPHSL, isolates have been routinely serotyped using both the Quellung method (SSI Diagnostica, Copenhagen, Denmark) and the sequential multiplex PCR (11). Thirty-one isolates collected over various years, for which serotypes could not be unambiguously determined by these methods, were serotyped using PCRSeqTyping (12) at the Laboratory of Life Sciences Center of Vilnius University (Lithuania). Isolates that could not be serotyped by either the Quellung method or PCR, and were negative for the *cpsB* gene but positive for *lytA* and *piaA*, were defined as non-typeable (NT) pneumococci. Detection of the *lytA* and *piaA* genes was conducted by conventional PCR using primers described in (13) and (14), respectively.

All isolates were recovered from blood or cerebrospinal fluid. Information on individuals' age was provided by the NPHSL. Data on pneumococcal vaccination coverage in children and adults were obtained from the National Public Health Center (15). Annual population data were retrieved from the Lithuanian Department of Statistics (https://osp.stat.gov.lt/).

### 2.2 Statistical analysis

Descriptive statistics were used to summarize the distribution of *S. pneumoniae* serotypes across different age groups and time periods. Serotype prevalence was expressed as absolute counts and percentages within defined time frames: the pre-PCV10 period (2010–2014) and the post-PCV10 period, which was further divided into three subperiods (2015–2017, 2018–2020, and 2021–2024). For trend analysis, serotypes were grouped into those covered by PCV10 (1, 4, 5, 6B, 7F, 9V, 14, 18C, 19F, and 23F), PCV13 (PCV10 serotypes plus 3, 6A, 19A), and non-vaccine serotypes. The study population was divided into children (< 18 years) and adults (≥18 years), with adults further stratified into age groups: 18–64 and ≥65 years.

Comparisons of serotype prevalence between periods (pre-vaccine vs. post-vaccine) and between post-vaccine subperiods were performed using Fisher's exact test, which is appropriate for small sample sizes and categorical data. For all comparisons, a two-sided *p*-value < 0.05 was considered statistically significant. Statistical analyses were performed using automated computational tools based on Python (version 3.11), SciPy, and Pandas.

### 2.3 Ethics statement

This retrospective study did not involve human subjects but analyzed microbial isolates. According to Lithuanian law, such research does not fall within the scope of biomedical research and does not require approval from the national Bioethics Committee. All patient-related data were anonymized using a unique coding system approved in the NPHSL. Information on individuals' ages and the date of strain isolation was provided following the Agreement between Vilnius University and NPHSL (No SU-237, 2025-01-30).

## 3 Results

### 3.1 Epidemiological background

According to the laboratory-based EU surveillance case definition for IPD, a case is defined as a person whose specimen, obtained from a normally sterile site, tests positive for *S. pneumoniae* by culture or PCR (16). However, based on this definition, adopted in clinical guidelines for IPD in Lithuania, the reported epidemiological data on disease cases remain inconsistent (Table 1) as the number of non-duplicate *S. pneumoniae* isolates obtained from normally sterile sites significantly exceeded the annually reported IPD cases during the periods 2010–2016 and 2021–2022 (17). Notably, during the COVID-19 pandemic (2019–2020), no IPD cases were reported to the ECDC from Lithuania. The number of IPD cases diagnosed by PCR without culture confirmation remains unknown. Due to limitations within the surveillance system, the reported IPD incidence likely underestimates the true burden of the disease in Lithuania.

**Table 1:** Distribution of invasive pneumococcal isolates by year and age group in Lithuania, 2010–2024.

**Year**	**2010–2012**	**2013–2014**	**2015**	**2016**	**2017**	**2018**	**2019**	**2020**	**2021**	**2022**	**2023**	**2024**
**Period**	**Pre-vaccine**						**Post-vaccine**					
IPD cases reported to the ECDC	25	23	25	56	76	65	0	0	25	88	142	No data
**Total number of isolates**	**55**	**99**	**83**	**94**	**90**	**61**	**84**	**79**	**81**	**144**	**157**	**163**
**Isolates by age**												
< 1 year	6	2	2	1	2	1	2	1	1	2	0	0
1–4 years	2	11	7	3	5	4	3	3	0	5	1	3
5–18 years	3	7	3	3	4	1	1	3	4	4	1	1
**Total children** (< 18 years)	**11**	**20**	**12**	**7**	**11**	**6**	**6**	**7**	**5**	**11**	**2**	**4**
18–44 years	8	22	14	17	10	7	11	4	10	13	21	23
45–64 years	27	33	27	17	25	15	30	22	25	46	43	55
>65 years	8	23	27	38	33	30	33	37	25	54	67	77
**Total adults** (≥18 years)	**43**	**78**	**68**	**72**	**68**	**52**	**74**	**63**	**60**	**113**	**131**	**155**
Age unknown	1	1	3	15	11	3	4	9	16	20	24	4

Data on PCV10 coverage in children (< 18 years) have been available since 2018, with coverage rates for at least one dose ranging between 80 and 85% within the NIP between 2018 and 2024 (15). Information on the PCV13 vaccination status of at-risk children is not available. In adults (≥18 years), vaccination coverage data have been available since 2020. Coverage with at least one dose of PCV13 or PPV23 among the adult population was 1.16% in 2020 and 1.19% in 2024, with lower coverage recorded in 2021 and 2022 (15).

### 3.2 Pneumococcal isolates and invasive serotype distribution in the overall population

Between 2010 to 2024, the NPHSL received a total of 1,190 invasive pneumococcal isolates for serotyping (Table 1). Of these, 154 isolates were collected during the pre-vaccine period (2010–2014) and 1,036 during the post-vaccine period (2015–2024). Most isolates originated from adult patients, accounting for 121 (78.6%) in the pre-vaccine period and 856 (82.6%) in the post-vaccine period. Isolates from children comprised 31 (20.1%) and 71 (6.9%) cases in the respective periods, while isolates with missing age information accounted for 1.3 and 10.3%, respectively.

The number of isolates per 100,000 population increased steadily, from 0.68 in 2012 to 1.89 in 2014, 3.32 in 2017, and reached 5.78 in 2024, following a decade of PCV10 implementation. This increase was most pronounced in adults aged ≥65 years, whose proportion of total isolates rose from 20% in 2010–2014 to 36% in 2015–2017, 44.5% in 2018–2020, and 41% in 2021–2024 (Table 1). Among adults aged 45–64 years, the proportion fluctuated from 39% in 2010–2014 to 26 % in 2015–2017 and 31% in 2021–2024. In contrast, the proportion of isolates from adults aged 18–44 years showed a slight decline, from 20% in the pre-vaccine period to 15% in 2015–2017 and 13% in 2021–2024. The annual number of isolates from children remained relatively low across the pre- and post-vaccine periods; however, compared to the pre-vaccine period, the proportion of IPD isolates originating from children decreased by approximately half.

**Figure 1:**
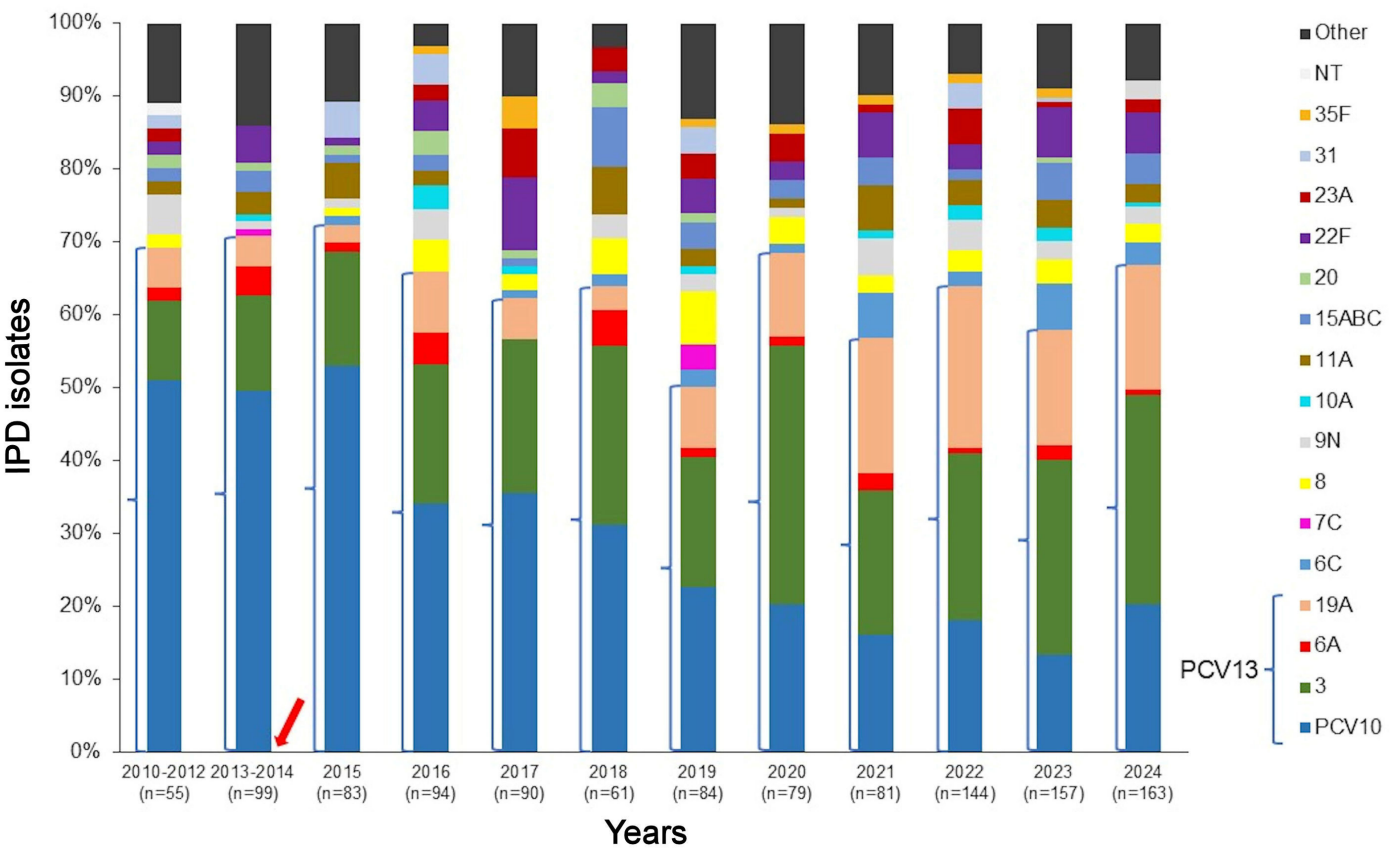
Distribution of invasive *S. pneumoniae* isolates in the overall Lithuanian population, 2010–2024. Only serotypes with a frequency exceeding 3% in at least one period are shown across all time periods. Serotypes covered by PCV13 are bracketed. The arrow indicates the introduction of PCV10 into the NIP in October 2014. NT, non-typeable isolates. Other serotypes: 6D, 9A, 9L, 18B/C/F, 11B, 12F, 13, 15F, 19C, 21, 23B, 24A, 25A/F, 28A/F, 29, 33F, 34, 35A/B/C, 38, 48.

The distribution of invasive *S. pneumoniae* serotypes in the overall Lithuanian population during the pre- and post-PCV10 periods is presented in Figure 1. A total of 40 different IPD serotypes were identified in the pre-vaccine period, compared to 58 serotypes in the post-vaccine period. Statistical analysis revealed a significant decline in PCV10 serotypes from 2010 to 2024 (*p* = 0.00002), with their proportion among total isolates decreasing from 50% in 2010–2014 to 24% in 2018–2020 and 17% in 2021–2024. Meanwhile, several non-PCV10 serotypes emerged or increased over time. Specifically, serotypes 19A (*p* = 0.0015) and 6C (*p* = 0.0061) demonstrated statistically significant rising trends. The proportion of 19A increased steadily from 4.5% in the pre-PCV10 period to 8.3% in 2019, reaching 22.2% in 2022. Serotype 3 cases of invasive disease showed a statistically significant rise over the 2010–2024 period, with an average annual increase of 1.33% (*p* = 0.004). Although serotype 22F exhibited a gradual increase, this trend was not statistically significant (*p* = 0.289). Other non-vaccine serotypes, such as 23A and 35F, were detected more frequently in later years; however, most did not reach statistical significance, likely due to small case numbers or variability across years.

### 3.3 Invasive pneumococcal serotype distribution by age groups

During the pre-vaccine period, a diverse range of serotypes caused IPD in children, with serotype 14 (22.5%) and serotypes 6A plus 6B (19.4%) being the most prevalent (Table 2). Serotype 6B was the most common among the carried serotypes in young children before vaccine implementation, accounting for 15.8%, while serotypes 14 and 6A were less frequently detected (9). Following the introduction of PCV10, the proportion of pneumococcal isolates covered by the vaccine declined significantly (*p* = 0.032), dropping from 17 isolates in 2010–2014 to just one isolate in 2021–2024. Notably, PCV10-covered serotype 14 showed a statistically significant decrease in the later post-vaccine subperiods, being absent in both 2018–2020 (*p* = 0.035) and 2021–2024 (*p* = 0.033) compared to seven cases in the pre-vaccine period. Among non-PCV10 serotypes, serotype 19A increased markedly, rising from near absence in the early years to nine isolates in 2021–2024 (*p* = 0.00033). Serotype 3 exhibited no statistically significant change over time. No other individual serotype demonstrated a significant shift across the study period. Interpretation of these findings is limited by the relatively small number of isolates, which may have reduced the power to detect more subtle changes in serotype distribution.

**Table 2:** Invasive *S. pneumoniae* serotypes isolated from children in Lithuania, 2010–2024.

**Serotypes**	**2010–2014**	**2015–2017**	**2018–2020**	**2021–2024**
	**Pre-vaccine**	**Post-vaccine**		
**Covered by PCV10**				
1	1 (3.2%)	0 (0%)	0 (0%)	0 (0%)
4	0 (0%)	1 (3.3%)	0 (0%)	0 (0%)
5	0 (0%)	0 (0%)	0 (0%)	0 (0%)
6B	2 (6.5%)	4 (13.3%)	2 (10.5%)	0 (0%)
7F	0 (0%)	0 (0%)	0 (0%)	0 (0%)
9V	1 (3.2%)	3 (10%)	0 (0%)	0 (0%)
14	7 (22.6%)	4 (13.3%)	0 (0%)	0 (0%)
18C	2 (6.5%)	4 (13.3%)	1 (5.3%)	0 (0%)
19F	2 (6.5%)	0 (0%)	0 (0%)	1 (4.5%)
23F	2 (6.5%)	2 (6.7%)	0 (0%)	0 (0%)
**Covered by PCV13 (additionally)**				
3	2 (6.5%)	2 (6.7%)	5 (26.3%)	5 (22.7%)
6A	4 (12.9%)	1 (3.3%)	1 (5.3%)	1 (4.5%)
19A	1 (3.2%)	2 (6.7%)	0 (0%)	9 (40.9%)
**Non-PCV10/PCV13 serotypes**				
9N, 12F, 18A, 23B	7 (22.6%)			
8, 9A, 9N, 10A, 15B, 18F, 35F		7 (23.3%)		
10A, 11A, 15B, 22F, 23A, 33F, 38			10 (52.6%)	
6C, 11A, 15C, 22F, 23B, NT				6 (27.3%)

**Figure 2:**
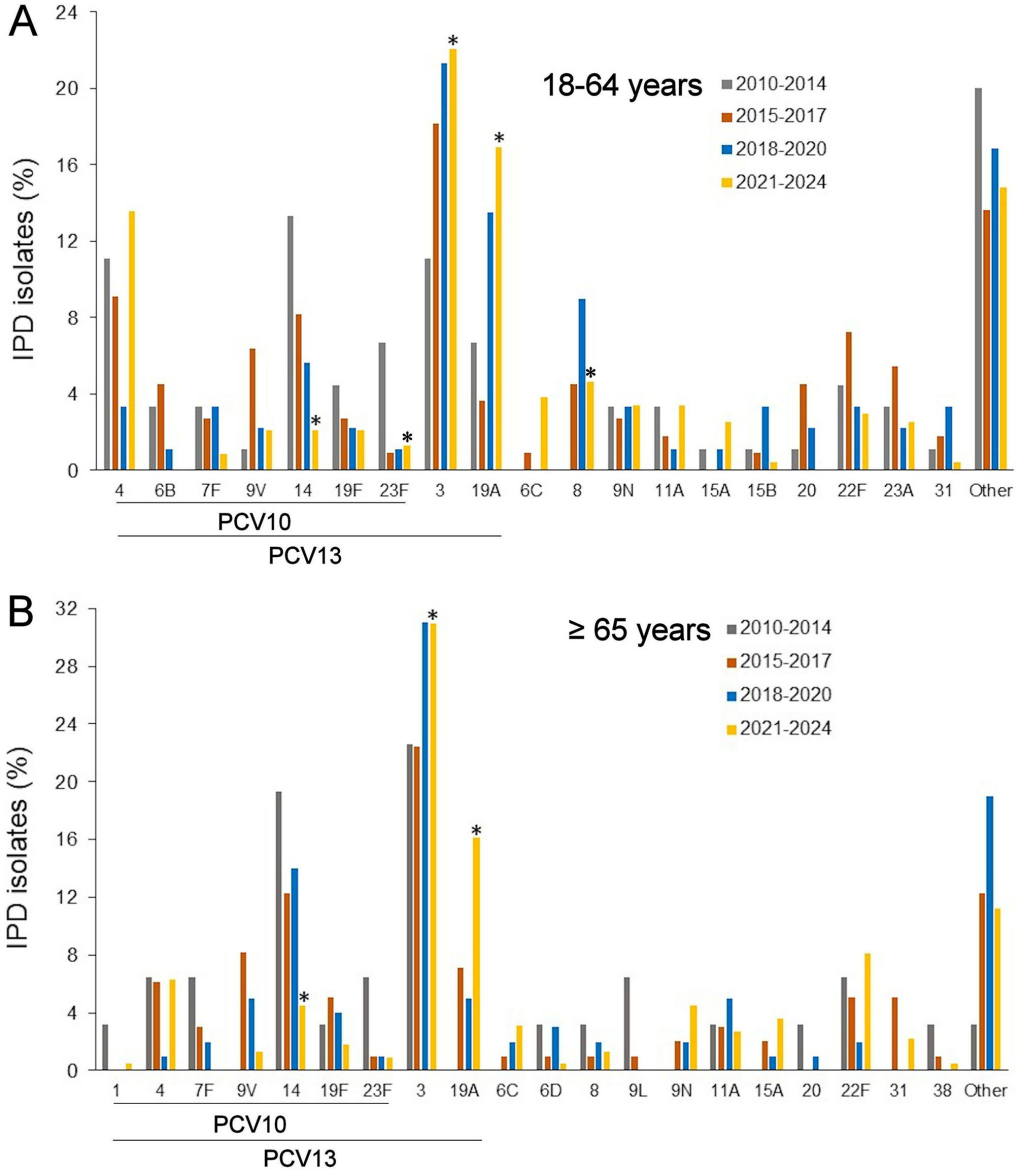
Invasive pneumococcal serotype distribution among adults aged 18–64 years **(A)** and ≥65 years **(B)** during the pre-vaccine period (2010–2014) and the post-vaccine subperiods (2015–2017, 2018–2020, 2021–2024). Only serotypes with a frequency exceeding 3% in at least one period are shown across all time periods. *Statistically significant difference compared to the pre-vaccine period (2010–2014), *p* < 0.05. Serotypes covered by PCVs are indicated. Other serotypes in **(A)**: 1, 7C, 6A, 9A, 9L, 10A/B, 11B, 12F, 15C/F, 17A/F, 18A/F, 21, 23B, 25A/F, 28A/F, 29, 33F, 34, 35A/B/F, 38, non-typeable. Other serotypes in **(B)**: 6A/B, 7C, 9A, 10A/F, 12F, 15C, 16F, 17A/F, 18C, 19C, 23A/B, 24A, 35B/F, 48, non-typeable.

The analysis of *S. pneumoniae* isolates among adults aged 18–64 years revealed substantial changes in serotype distribution following the introduction of PCV10 (Figure 2A). The proportion of isolates corresponding to PCV10 serotypes decreased significantly, from 45.6% in 2010–2014 to 22.0% in 2021–2024 (*p* = 0.00006). Notably, the prevalence of serotype 14 declined from 13.3% in 2010–2014 to 2.1% in 2021–2024 (*p* = 0.0002). Serotype 23F also decreased from 6.7 to 1.3% (*p* = 0.015). Other PCV10 serotypes, including 4, 7F, 9V, and 19F, exhibited modest fluctuations in frequency, but these changes were not statistically significant. In contrast, the prevalence of non-PCV10 serotype 19A increased markedly, from 6.7% in 2010–2014 to 16.9% in 2021–2024 (*p* = 0.020). The prevalence of serotype 3 did not demonstrate a statistically significant change between the pre-vaccine period and the early post-vaccine periods (2015–2017, OR = 0.56, *p* = 0.232; 2018–2020, OR = 0.46, *p* = 0.071). However, a statistically significant increase was observed in the later post-vaccine period (2021–2024, OR = 0.44, *p* = 0.027), indicating a rising trend of serotype 3 disease in this age group. Additionally, serotype 8, which was absent in the pre-vaccine period, emerged in the post-vaccine period, accounting for 4.7% of cases (*p* = 0.039).

In adults aged ≥65 years, the proportion of PCV10 serotypes decreased significantly from 45.0% in 2010–2014 to 15.2% in 2021–2024 (*p* = 0.0006; Figure 2B). Serotype 14 demonstrated a statistically significant decline from 19.4% in the pre-vaccine period (2010–2014) to 4.5% in 2021–2024 (*p* = 0.009). Other PCV10 serotypes, including 4, 7F, and 9V, did not show statistically significant changes, although fluctuations in frequency were observed. Due to small isolate counts, formal statistical testing was not performed for several lower-frequency serotypes. In contrast, isolates corresponding to additional PCV13 serotypes, specifically serotypes 3 and 19A combined, increased significantly over the study period, from seven isolates (22.5%) in 2010–2014 to 105 isolates (47.0%) in 2021–2024 (*p* = 0.0049). However, when assessed individually, serotype 3 did not demonstrate a statistically significant increase (*p* = 0.31), while serotype 19A exhibited a substantial rise over the same period (*p* = 0.0066). The proportion of non-PCV10 serotypes did not change significantly (*p* = 1.000), despite an absolute increase in case numbers.

Within the post-vaccine period (2015–2024), most serotypes exhibited no statistically significant trends across the subperiods. Serotype 4 showed a borderline significant decrease between 2015–2017 and 2018–2020 (*p* = 0.064); however, this trend was not sustained in subsequent years. Overall, the limited number of isolates for most individual serotypes reduced the statistical power to detect intra-decade changes.

Analysis of pneumococcal serotype distribution in the 2021–2024 period revealed several significant differences between adults aged 18–64 years and those aged ≥65 years. Serotypes 4 and 8 were significantly more prevalent among individuals aged 18–64 years (*p* = 0.0045 and *p* = 0.031, respectively). In contrast, serotype 22F was significantly more common in adults aged ≥65 years (*p* = 0.037). No other serotype showed statistically significant differences between the age groups; however, some low-prevalence serotypes could not be analyzed due to limited sample sizes.

## 4 Discussion

This study provides the first comprehensive analysis of IPD serotype distribution in Lithuania over a 15-year period, covering both the pre-vaccine phase and a decade following vaccine implementation. After the nationwide implementation of PCV10 for childhood immunization in 2014, a marked decline in PCV10 serotypes was observed across the entire population, consistent with trends reported in other countries after vaccine introduction (18–20). In children, the significant decline provides strong evidence of direct vaccine impact. Among PCV10 serotypes, the most notable decrease was observed in serotype 14, which had been one of the most common causes of IPD in both children and adults. Notably, serotype 5 was not detected among IPD isolates throughout the study period.

Despite the reduction in vaccine serotypes, the overall number of IPD isolates increased during the post-vaccine years, particularly among older adults. This rise likely reflects ongoing serotype replacement and, in our view, the improved diagnostic practices. In our study, several non-PCV10 serotypes emerged or increased over the post-vaccine period, most notably serotypes 19A, 6C, and 3. In children, the rise of serotype 19A was also observed, whereas serotype 3 was detected across all periods; its frequency remained low, and changes were not statistically significant. Among adults, serotype replacement appears to be more pronounced, as evidenced by the increased prevalence of serotypes 3 and 19A, and a rising incidence of serotypes such as 6C, 8, and 22F. Interestingly, a rise in serotype 8 was observed in both children and adults, primarily in countries using PCV13 (21, 22), however, this serotype was also prevalent among adults aged 18–64 years in Lithuania during the post-PCV10 period. Among serotypes not included in either PCV10 or PCV13, an increase in serotype 22F was also detected among individuals aged ≥65 years in European countries (23, 24). An increase in serotype 6C has been observed in countries that implemented PCV10, but not PCV13, likely due to a cross-protection with 6A, which is included in PCV13 (23). Serotype 3 has remained the most prevalent IPD serotype since 2015 in Lithuania. Numerous reports reflected suboptimal PCV13 protection against this serotype, thereby highlighting the need for enhanced strategies to address it (25, 26).

In Lithuania, serotype 19A has shown a steadily increasing trend since 2015, peaking in 2021. It became the second most prevalent IPD serotype among adults aged 18–64 during the 2018–2024 period, and among those aged ≥65 during 2021–2024. However, its increase among children was less apparent, due to the low number of pediatric isolates, which may obscure the true incidence of IPD serotypes in this age group.

The substantial increase in serotype 19A, which is included in PCV13 but not in PCV10, aligns with findings from previous studies conducted in countries that implemented PCV10 (18, 19, 27). The rise in 19A is concerning due to its high invasiveness and documented association with antibiotic resistance, as reported in neighboring Latvia (28) and more distant countries (24, 29). Importantly, the implementation of PCVs has influenced *S. pneumoniae* antibiotic resistance patterns, an effect that should be considered when selecting empiric treatment for pneumococcal disease (27, 28, 30). The most recent study on the antibiotic resistance of pneumococcal isolates in Lithuania was conducted by the research group before the introduction of PCV10 (31). However, antimicrobial resistance is not included in standard surveillance protocols, a limitation that persists to this day and weakens the relevance of empiric treatment options.

In Lithuania, the low vaccination coverage with vaccine PCV13 did not lead to a reduction in 19A IPD, unlike in other countries where indirect vaccine effects were observed in older age groups following the introduction of PCV13 into childhood immunization programs (23, 26, 32). PCV13 elicits an immune response against serotype 19A, likely reducing nasopharyngeal carriage in vaccinated children and subsequently decreasing transmission, thereby providing herd protection to unvaccinated children and adults (32). Of particular concern is the growing burden of IPD in adults aged ≥65 years in Lithuania. The low adult vaccination rate and serotype replacement contribute to sustained transmission and disease in this high-risk group. The introduction of PCV20 for adult immunization in Lithuania in 2024 is expected to provide broad serotype coverage, including serotypes that have emerged due to serotype replacement following the implementation of PCV10. Notably, PCV20, but not PCV15, includes serotype 8, which has shown an increasing trend in IPD cases among adults in Lithuania. However, cost-effectiveness models evaluating the use of higher-valency PCVs in older populations, such as those conducted in other countries, have demonstrated that close monitoring of childhood and adult immunization with PCV15 and PCV20 is essential to effectively reduce the burden of IPD (33, 34).

Limitations in IPD surveillance have hindered the ability of Lithuanian health policymakers to make timely, evidence-based decisions regarding invasive pneumococcal serotype distribution and the impact of PCVs. Our findings showed that serotype 19A and, in particular, serotype 3 accounted for a substantial proportion of IPD cases and exhibited an increasing trend during the pre-vaccine period, suggesting that the introduction of PCV13, rather than PCV10, in 2014 may have provided broader protection against circulating serotypes. In Belgium, the timely detection of a significant increase in pneumococcal carriage of serotypes 19A and 6C in children following the switch from PCV13 to PCV10 prompted a return to PCV13, which subsequently led to a reduction in serotype 19A carriage among children (35, 36). This experience highlights the importance of monitoring pneumococcal serotype dynamics through carriage studies in the post-vaccination period, an approach that has not yet been implemented in Lithuania.

Due to underreporting of IPD in Lithuania, the national surveillance system underestimates the true burden of disease, complicating vaccination policy decisions and hindering efforts to prevent severe disease outcomes and protect lives. Incomplete data on the clinical presentation of IPD limit the ability to assess the impact of pneumococcal serotypes and antibiotic resistance patterns on the most severe forms of the disease. For many years in Lithuania, infectious diseases were not prioritized by healthcare policymakers, which adversely affected the development and effectiveness of national surveillance systems. Several factors continue to limit the efficacy of the surveillance system, including weakness in organizational structure, insufficient funding, limited collaboration with scientific experts, and inconsistent participation of medical doctors and clinical microbiologists in isolating and submitting pneumococcal strains to the NPHSL. Additionally, inadequate laboratory capacity for strain isolation and identification in some regional healthcare settings further hampers effective surveillance.

Underreporting in the IPD surveillance system may have affected our study findings, potentially limiting the accuracy of vaccine impact estimates. Additionally, the small number of pediatric isolates, particularly in the later years, limits the generalizability of the results for this age group. Nevertheless, despite these limitations, the study represents the first comprehensive assessment in Lithuania of the impact of vaccination on the serotype structure of invasive pneumococcal isolates.

## 5 Conclusion

Our study provides evidence of an indirect effect of the childhood PCV10 vaccination in the adult population in Lithuania, most of whom did not receive the vaccine. These findings underscore the importance of ongoing pneumococcal serotypes surveillance to monitor the emergence and expansion of non-vaccine serotypes, such as 19A, which are included in higher-valency vaccines and pose a particular risk to vulnerable populations, especially older adults. Furthermore, long-term surveillance studies, such as ours, are essential for assessing whether the maximum impact of implemented vaccination programs has been achieved and for informing timely adjustments to immunization strategies. Additionally, future studies incorporating genetic tools are essential for tracking the pneumococcal lineages associated with emerging and epidemic-prone serotypes.

## Data Availability

The original contributions presented in the study are included in the article/supplementary material, further inquiries can be directed to the corresponding author.
